# Specificity Effects of the Coupling Auditory Rhythm on Neural Oscillations of Older Adults

**DOI:** 10.1093/geroni/igaf122.2036

**Published:** 2025-12-31

**Authors:** Yilin Chen, Tommy Lam, Davynn G H Tan, Karen P Y Liu

**Affiliations:** The Hong Kong Polytechnic University, Hong Kong, China (People’s Republic); The Hong Kong Polytechnic University, Hong Kong, Hong Kong, China (People’s Republic); The Hong Kong Polytechnic University, Hong Kong, Hong Kong, China (People’s Republic); The Hong Kong Polytechnic University, Hong Kong, Hong Kong, China (People’s Republic)

## Abstract

Sensory entrainment may enhance cognitive performance by modulating abnormal neural oscillations associated with cognitive impairment, potentially alleviating healthcare burdens. The study investigated the effectiveness of different auditory rhythms in altering neural oscillations in the aging brain. We compared changes (in mean power spectral density [PSD]) within the frontal and parietal lobes in response to the single rhythm (3 or 40 Hz.) and coupling rhythm (3 and 40 Hz. coupling). Twelve participants (69.17 ± 0.79 years) underwent electroencephalography recordings during three rhythm conditions in one session. Each condition included a 120-second auditory exposure state, with 30-second pre- and post-exposure resting states. Conditions were randomly ordered and blinded to participants. PSD during exposure was normalized by subtracting the pre-exposure PSD. The 3 Hz. PSD change was significantly higher for 3 Hz. condition (mean: 3.58 dB, range: -2.24 to 10.60) compared to the coupling condition (mean: 1.91 dB, range: -5.01 to 11.42) at the parietal lobe (p < 0.001). The 40 Hz. PSD change was significantly lower for the 40 Hz. condition (mean: -0.45 dB, range: -11.06 to 4.32) compared to the coupling condition (mean: 1.40 dB, range: -17.90 to 9.58) at the frontal lobe (p = 0.014). The coupling rhythm showed stronger effectiveness on 40 Hz. PSD but weaker effects on 3 Hz. PSD, demonstrating the specific effects on 40 Hz. PSD. This provides a novel approach to enhance targeted neural oscillations in aging. Further studies should investigate the long-term effects of oscillation modulation on various cognitive functions in the elderly.

